# Biological Role of Folic Acid in Pregnancy and Possible Therapeutic Application for the Prevention of Preeclampsia

**DOI:** 10.3390/biomedicines11020272

**Published:** 2023-01-19

**Authors:** Lyazzat Kaldygulova, Talshyn Ukybassova, Gulzhanat Aimagambetova, Andrey Gaiday, Akylbek Tussupkaliyev

**Affiliations:** 1 Department of Obstetrics and Gynecology #2, West-Kazakhstan Marat Ospanov Medical University, Aktobe 030012, Kazakhstan; 2 Clinical Academic Department of Women’s Health, CF “University Medical Center”, Astana 010000, Kazakhstan; 3 Department of Biomedical Sciences, School of Medicine, Nazarbayev University, Astana 010000, Kazakhstan

**Keywords:** folic acid, folate, MTHFR, homocysteine, pregnancy, preeclampsia

## Abstract

The rationale and importance of folic acid supplementation during pregnancy for fetal congenital defect prevention are accepted worldwide. Moreover, a sufficient plasma concentration of folates can reduce the incidence of spontaneous abortions, and support the normal expansion of placental blood vessels, ensuring physiological placental blood flow, thus promoting appropriate fetal growth and development. Furthermore, there is emerging evidence that long-term supplementation with folic acid can effectively prevent preeclampsia. Preeclampsia is unique to the human species in complications during pregnancy, which contributes to maternal and perinatal mortality worldwide. In the pathogenesis of preeclampsia abnormal placental invasion, the excess of antiangiogenic factors and maternal–placental syndrome play a key role. Increased blood levels of homocysteine during pregnancy are associated with the risk of preeclampsia. Moreover, hyperhomocysteinemia has been proposed to be an independent risk factor for preeclampsia. Folate supplementation helps to decrease elevated levels of homocysteine; thus, the role of folic acid supplementation in pregnancy is even more important. Multiple reports suggest that folate administration decreases the level of serum homocysteine and, therefore, reduce the risk and severity of preeclampsia. However, the association between folic acid supplementation and the decreased risk of preeclampsia has been investigated with controversial conclusions. Currently, the optimal dose of folic acid that is effective for preeclampsia prevention remains uncertain. In this review, we aim to summarize the accumulated knowledge on the role of folic acid in the pathogenesis of preeclampsia, and the possible impact of folate supplementation on the decreased risk of preeclampsia.

## 1. Introduction

Preeclampsia is one of the most serious complications during pregnancy leading to multiple maternal and perinatal complications [1], and it is becoming progressively more common in developed countries [2]. Preeclampsia remains a common cause of maternal and fetal morbidity and mortality [1,2,3,4]. A worldwide trend in delaying childbearing, especially in high-income countries, contributes to the risk factors associated with preeclampsia, which include advanced maternal age, obesity, insulin resistance, and other somatic conditions [2]. Inadequate or the lack of prenatal care in part explains the high and increasing prevalence of preeclampsia in developing countries [1,2,3].

The exact etiological factors of preeclampsia remain unclear. However, it is believed that two crucial steps play an important role in the pathogenesis of preeclampsia: abnormal placentation followed by maternal–placental syndrome related to an excess of antiangiogenic factors [1,2,5,6]. These currently proven hypotheses on preeclampsia as a placental disease help in the management of the related manifestations.

Preeclampsia is considered a uniquely challenging condition due to existing questions about its etiology, pathogenesis, and therapeutic management that simultaneously affect the mother and fetus [6]. Multiple research studies have been conducted to investigate the possible preventative and treatment options for preeclampsia [1,3,7,8,9]. Accumulating evidence has suggested that folic acid metabolism abnormalities and elevated levels of blood homocysteine contribute to the development of hypertensive disorders in pregnancy, including preeclampsia [8,10,11]. Many studies confirm that folate administration can help to reduce elevated blood homocysteine levels [8,12,13,14,15,16,17,18]. However, the association between folic acid supplementation and decreased risk of preeclampsia has been investigated with inconsistent conclusions and different suggested doses [7,8,19,20,21,22,23,24]. Whether folate administration in pregnancy can contribute to the prevention of preeclampsia remains uncertain [7].

Thus, in this review, we aim to summarize and update the accumulated knowledge on the role of folic acid in the pathogenesis of preeclampsia, and the possible impact of folate supplementation on the decreased risk of preeclampsia. A better understanding of folic acid metabolism in preeclampsia might open new opportunities for the prevention of preeclampsia and related adverse pregnancy outcomes.

## 2. Materials and Methods

A non-systematic literature review was performed by the authors, searching the available data on folic acid supplementation in pregnancy and the possible effects for preeclampsia prevention. The literature search was conducted in Scopus, Web of Science, PubMed, EBSCO, and Google Scholar databases up to the current time (2022), using the following keywords, combinations of keywords, and MeSH IDs (if available): “folate”, “folic acid” (MeSH Unique ID: D005492), “pteroylpolyglutamic acids” (MeSH Unique ID: D011624), “tetrahydrofolates” (MeSH Unique ID: D013763), “folic acid deficiency” (MeSH Unique ID: D005494), “folate metabolism”, “methylenetetrahydrofolate reductase”, “MTHFR” (MeSH Unique ID: D042965), “MTHFR deficiency”(MeSH Unique ID: C565512), “methylenetetrahydrofolate reductase deficiency” (MeSH Unique ID: C537357), “homocysteine” (MeSH Unique ID: D006710), “hyperhomocysteinemia” (MeSH Unique ID: D020138), “hypertension, pregnancy-induced” (MeSH Unique ID: D046110), “preeclampsia” (MeSH Unique ID: D011225), “preeclampsia prevention”, “obstetrical complication” (MeSH Unique ID: D007744), “prognosis” (MeSH Unique ID: D011379). The most relevant to the subject of the discussion, peer-reviewed publications have been read and employed for the review based on the authors’ evaluations. The results of the research have been divided into different sections and subsections in a logical sequence to illustrate what has been reported on the topic of discussion.

## 3. Results and Discussion

### 3.1. Folic Acid

Folic acid (pteroylglutamic acid) is one of the B-group vitamins important for cell development [4,25,26,27,28,29]. The term ‘folate’ covers a number of various forms, all of which contain a pteroyl group [30]. Natural folate (Vitamin B9) is a water-soluble substance that exists as tetrahydrofolate and methyltetrahydrofolate [4,30]. Folic acid is a synthetic, artificial substance widely used for food fortification and as a dietary supplement [4,27,30]. Folates can be found in high concentrations in the liver and in vegetables [29].

Folic acid and its role in health were first recognized in 1930 by Lucy Wills [25,28,31]. Later, in 1945, folic acid was successfully synthesized in the form of pteroylmonoglutamate with a therapeutic activity equal to the natural form [25,32]. Folate deficiency is closely linked to elevated plasma homocysteine, which is a serious risk factor for cardiovascular complications and stroke [14,27,29,33]. Additionally, a low folic acid status is associated with the “altered methylation of DNA that may affect gene expression and uracil-induced genomic instability” [27]. These metabolic changes may increase the risk of cancer, Alzheimer’s disease, and cognitive dysfunction [25,27,29,33].

In modern medicine folic acid is recognized as a major component of the periconceptional care for women [8,25,27,29]. A deficiency in folic acid can lead to fetal congenital defects and megaloblastic anemia in the mother [25,28,29]. Currently, the World Health Organization (WHO) recommends 400 µg daily folic acid supplementation [34,35]. Thus, many countries have created national programs for mandatory folic acid food fortification to fight inadequate dietary folate consumption, and to reduce the prevalence of folate-related diseases [27,29].

#### 3.1.1. Folic Acid Metabolism

In the human body, folate is absorbed in the mucosal epithelial cells [27,29,36]. A folylpoly-glutamate carboxypeptidase enzyme plays a primary role in this physiologic process, which results in monoglutamate forms [27,29,36]. After ingestion, most folic acid is absorbed in the upper part of the small intestine by a specific pH- and energy-dependent transport mechanism [27,36,37]. Once folic acid and derivatives appear in the enterocytes and are metabolized to 5-methyltetrahydrofolate (5-MTHF), it is exported into the portal vein [29]. The liver has a high affinity for folic acid but a lower affinity for the removal of 5-methyltetrahydrofolate (5-MTHF) [27]. This fact allows a fraction of the 5-MTHF to proceed without obstacles into the systemic circulation [27,29,36,37].

**Figure 1:**
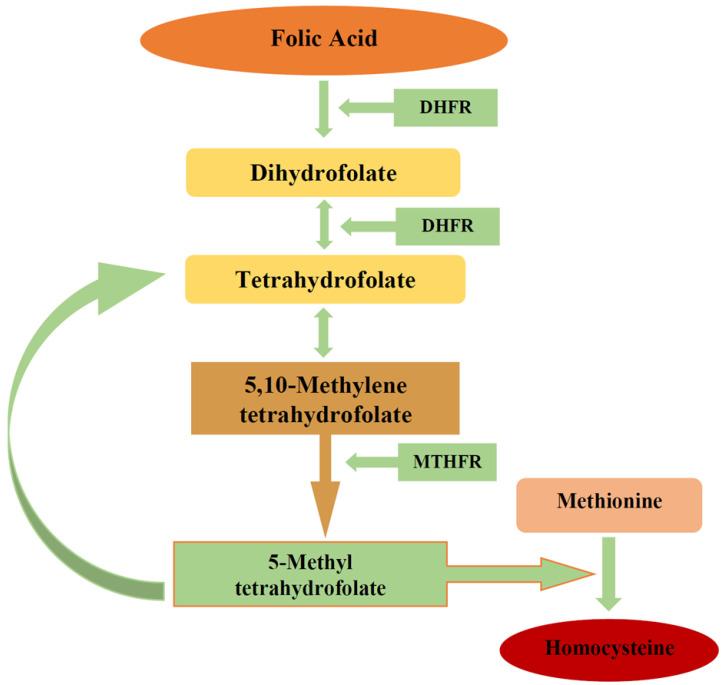
Folic acid metabolism. Figure legend: DHFR—dihydrofolate reductase; MTHFR—methylenetetrahydrofolate reductase.

Further, some of the folate in hepatocytes is secreted in bile and then reabsorbed in the intestine [29]. The overall process of folic acid metabolism includes many steps and enzymes (Figure 1). Folic acid’s biological activity relies on the action of the dihydrofolate reductase (DHFR) enzyme produced in the liver [29,30]. In order to carry out its biological function, folic acid needs to be reduced by DHFR to dihydrofolate, and then to tetrahydrofolate [36]. Then, it is converted to its biologically active form 5-MTHF (Figure 1) [36].

The intestinal metabolism of folic acid to 5-MTHF is pH-dependent and according to studies appears to be extensive at pH 6.0 and minor at pH 7.5 [25,27,28]. Notably, high oral doses of folate (260–280 μg) have been reported to lead to the direct appearance of untransformed/unmetabolized folic acid in systemic circulation [27,29]. This indicates that intestinal conversion is not a mandatory premise for folate absorption and transport [27]. Thus, physiological and high doses of oral folic acid could pass through different metabolic pathways.

#### 3.1.2. Folic Acid Function

Folates are crucial to human cell development and function as a component and accelerator for essential biochemical reactions [4,30] and for cell multiplication and differentiation processes [38]. Folic acid plays an essential role in nucleotide metabolism important for DNA synthesis and methylation [25,27,30]. Folic acid and its derivatives function as acceptors or receptors for one-carbon units and function as co-enzymes involved in various methylation reactions [29,37].

Folates are also proposed to have important roles in other physiological processes required for a successful pregnancy, including antioxidant protection, angiogenesis, endothelial-dependent vascular relaxation, and homocysteine methylation [17,29,33,37,38,39]. These steps are important for chorion and placental development and the establishment of placental circulation, enabling fetal growth and development and favorable pregnancy outcomes [38].

Folate has different functions, including the metabolism of methionine to homocysteine (Figure 1), purine and pyrimidine synthesis, and DNA methylation [17,29,37,40]. Therefore, a low folate status might lead to chronic diseases caused by inadequate DNA synthesis and gene expression. Folate deficiency is associated with an increased risk of cardiovascular diseases, cancers, and cognitive dysfunction, including Alzheimer’s disease [17,27,29,33,39]. In addition, genetic polymorphisms of the methylenetetrahydrofolate reductase (MTHFR) enzyme result in elevated homocysteine levels and serves as an independent risk factor for folate-associated diseases [17,29,33,39].

Folate deficiency in the vast majority of cases occurs due to an inadequate dietary intake, which leads to decreased plasma folate levels followed by increased concentrations of homocysteine [17]. Moreover, low levels of folic acid and high concentrations of homocysteine in the blood are potential causes of neural tube defects [17].

#### 3.1.3. *MTHFR* Gene Physiology and Polymorphism

Another enzyme that plays an important role in folate metabolism is 5,10-methylenetetrahydrofolate reductase. The MTHFR enzyme is mapped to chromosome 1 (1p36.3) [15,41]. The enzyme functions in the reduction from 5,10-methylenetetrahydrofolate to 5-MTHF (Figure 1) [15,27,29,37]. The 5,10-MTHF conversion to 5-MTHF under MTHFR enables the production of methyl donors for the conversion of homocysteine to methionine [15]. Thus, MTHFR takes an essential role in homocysteine metabolism to methionine from 5-MTHF, which acts as a substrate for vitamin B12-dependent methioninesynthase (Figure 1) [15,17,29].

The *MTHFR* gene has been reported to possess one common mutation, *C677T*, linked to serious enzymatic deficiency and fourteen rare mutations, which are associated with severe enzymatic deficiency [15,16,41]. The *C677T* polymorphism is a point mutation at position 677 on the *MTHFR* gene characterized by the substitution of cysteine with thymine [15,42]. Following this mutation in the gene, alanine is substituted for valine, which leads to a deleterious effect on plasma homocysteine metabolism—high plasma homocysteine levels (hyperhomocysteinemia) and low folate levels [15,29,42,43]. Homocysteine is a homolog of cysteine (amino acid) with one additional methylene group and containing a sulfhydryl group [42,44]. The normal homocysteine levels range between 5 and 15 μmol/L [42,45]. Levels of 15 to 30 μmol/L are accepted as a mild increase, levels of 30 to 100 μmol/L reflect a moderate increase, while a value > 100 μmol/L is classified as severe hyperhomocysteinemia [42,45].

The prevalence of the *MTHFR 677C > T* varies depending on ethnic background and place of residence [15,43,46]. The allele frequency has been found to be higher in the Southern European population (Italian and Hispanics) and lower in Germans, and African Americans [15,46]. Among Caucasians living in Canada, the United States (US), Brazil, and Australia, the homozygous mutation is reported in up to 15% of the population [15,46]. Limited data are available about the Asian population. The Japanese population has been reported to have a homozygous mutation in 11–15% of the population [15,17]. Some limited reports are available from Central Asia. In Kazakhstan, low folate status and mild homocysteinemia were reported among the Kazakh population [16,18]. The cited study showed that *MTHFR C677T* mutation levels were high [16,18]. Similar findings were reported among the Kazakh ethnic minority in China, where low serum folic acid levels and high methylation of *MTHFR* gene were found [47,48,49,50]. A low frequency of the *MTHFR 677C > T* mutation was found in the sub-Saharan African population [15,51]. Due to the above-discussed metabolic pathways, when folic acid or vitamin B12 levels are inadequate, the metabolism of homocysteine is also affected, leading to hyperhomocysteinemia [15,29,30]. Polymorphism of the *MTHFR* gene at position *677C > T* may affect the enzyme’s role in the homocysteine pathway [15,16,17,18].

High plasma homocysteine levels have been proven to serve as a risk factor for multiple conditions such as atherosclerosis, coronary artery disease, essential hypertension, retinal vein occlusion, venous thromboembolism, cancers, infertility, and pregnancy complications (preeclampsia, recurrent pregnancy loss, etc.) [14,27,29,33,52,53,54]. Hyperhomocysteinemia has been reported in 40% of patients with coronary artery disease, and cerebral or peripheral artery disease [15,53]. The exact mechanism of how increased levels of homocysteine leads to the development of vascular endothelial pathology is still unknown. Some of the hypothesized mechanisms include hyperproliferation of vascular smooth muscle cells accompanied by endothelial dysfunction, oxidative damage, and a further increase in collagen synthesis, and damage to the arterial wall elastic layer [53].

The *MTHFR C677T* polymorphisms are associated with various diseases and pregnancy complications, including preeclampsia [15]. Thus, the role of folic acid supplementation in pregnancy is essential and attempts to find new applications of folates to prevent pregnancy-related conditions/complications are under investigation.

### 3.2. The Role of Folic Acid Supplementation in Pregnancy

Most women of childbearing age have no adequate dietary supply of folic acid [36,55,56,57]. Thus, the importance of folate administration during pregnancy for fetal congenital defect prevention is accepted worldwide [4,34,58]. An intake of 400 μg/day folic acid during the preconception period [34,55,58] and the first trimester of pregnancy ensures adequate folate blood levels during the organogenesis and decreases the risk of neural tube defects by 50–70% [4,34,36,56,57]. International guidelines recommend the administration of folic acid at doses between 400 and 800 μg/day [56,57] High-dosage folate supplementation (4–5 mg/day) should only be used for women at high risk, suffering from infertility, pregnancy loss, and previous newborns with neural tube defect [57].

Moreover, genetic polymorphisms of the MTHFR gene leading to hyperhomocysteinemia potentially could elevate the risk of congenital anomalies [4,36,55,56]. The effect of elevated homocysteine in combination with low levels of folic acid increases the risk of birth defects. To allow a sufficient supply of folate for persons with reduced MTHFR enzymatic activity, supplementation with a combination of folic acid and 5-MTHF can be performed [55,57]. Folic acid supplementation allows for the reduction of high blood levels of homocysteine [4,59], thus decreasing the risk of venous thromboembolic complications in pregnancy.

Inadequate folate supplementation has been associated with miscarriage, placental abruption, and intrauterine growth restriction (IUGR) [38,60,61]. Therefore, some studies have investigated the possibility of IUGR [60,62,63,64] and pre-term birth [63,65,66,67] prevention by the administration of folic acid [4]. However, the results are controversial: according to the limited reports, higher maternal folate levels could significantly lower the risk of pre-term birth [66,67], while other findings contrast this [65]. Some other researchers have reported an association between higher maternal levels of folate in early pregnancy with gestational diabetes mellitus risk [68]. Thus, since there are conflicting data on the positive effects of folic acid supplementation, neural tube defect prevention by daily folic acid supplementation remains the most important and proven intervention [4,65].

#### Folic Acid in the Fetus and Placenta

As the folic acid concentration in fetal blood flow is much higher than that in maternal, some researchers hypothesized that “folic acid intake during pregnancy could lead to the accumulation of inactive metabolites in fetal serum” [4]. In addition, a significantly higher concentration of 5-MTHF was reported in umbilical cord blood than in the corresponding mothers’ blood [4,69].

Results of an in vitro study demonstrated that the placental physiology and function may be compromised in conditions of folate deficiency, and “not necessarily” in conditions of folic acid excess [70]. However, other researchers report contrasting data after experimental investigation. In a recent experimental study Luan et al., (2021) proposed that “moderate increases” in folic acid intake during pregnancy may result in placental metabolic variations, leading to gene expression changes, specifically responsible for angiogenesis [4,71]. This may contribute to abnormal behavior in offspring [71]. The authors suggest determining a safe upper limit for folic acid supplementation during pregnancy [71]. Thus, further studies should aim to clarify the therapeutic window of folic acid intake during pregnancy.

### 3.3. Preeclampsia

#### 3.3.1. Epidemiology of Preeclampsia

Preeclampsia is a hypertensive disorder of pregnancy, which complicates up to 5–8% of pregnancies globally and is one of the major causes of maternal and perinatal mortality worldwide [1,3,6,8,9]. The prevalence of preeclampsia varies among different racial and ethnic groups by 7–11% [72]. This may be explained by racial and ethnic genetic structure variations. As was highlighted in a recent report, in the US, racial and ethnic minority groups, such as non-Hispanic Black women and American Indian women, are more affected by preeclampsia [72]. Studies report that human leukocyte antigen G (HLA-G) mutations may contribute to the development of preeclampsia [72,73,74,75]. This partially explains the increased risk of preeclampsia among women of African ancestry, as studies have demonstrated that the maternal HLA-G genotype is significantly associated with the risk of preeclampsia in African American women [72,76,77,78]. Furthermore, genetic variations in HLA-G may play a role in miscarriage and pre-term birth, which are known to be linked with HLA-G polymorphism [73,74,75].

In addition to maternal genetic variants that may predispose to preeclampsia, the fetal genome has also been found to be associated with the increased risk of preeclampsia [79,80,81]. Studies investigating the role of the fetal genome in the development of preeclampsia reported that dysregulation of the FMS-like tyrosine kinase 1 (FLT1) locus in the fetal genome is a fundamental molecular defect in preeclampsia [80], consequently, variants in the fetal genome near FLT1 are associated with an increased risk of preeclampsia [79,80,81]. Thus, more studies on factors leading to the lower or higher prevalence of preeclampsia among various ethnic groups could provide a better understanding of the pathogenesis of preeclampsia and ways to prevent it.

The prevalence of preeclampsia is of specific importance in controlling maternal mortality. Globally, more than 50% of maternal deaths in the period of 2003–2009 were due to hypertensive disorders, hemorrhage, and sepsis [82,83]. Causes of maternal mortality vary depending on the world region and a particular country’s income. The most frequent causes are postpartum hemorrhage and hypertensive disorders in pregnancy, with rates of mortality due to these complications varying from 36.9% in northern African countries to 16.3% in high-income countries [83]. Maternal deaths due to hypertension in pregnancy are most common in Latin America and the Caribbean, accounting for up to 26% of deaths [3,82,83]. Even in high-resource settings, in high-income countries, around 16% of maternal mortality cases are attributed to hypertensive disorders, while in low- and middle-income countries (LMICs) hypertensive disorders are responsible for up to 25% of maternal mortality cases [3,84]. Cases of hypertensive disorders in pregnancy, including preeclampsia, are increasing worldwide [2]. In the US, in the period of 17 years (1987–2004), the rate of preeclampsia increased by 25% [3,85].

Women with pregnancy complicated by preeclampsia may have infants with neurocognitive dysfunctions and suboptimal development in the offspring [1,2,8]. Hypertension and preeclampsia during pregnancy may also serve as risk factors for diabetes and cardiovascular disease in later life [8,9].

#### 3.3.2. Pathogenesis of Preeclampsia

**Figure 2:**
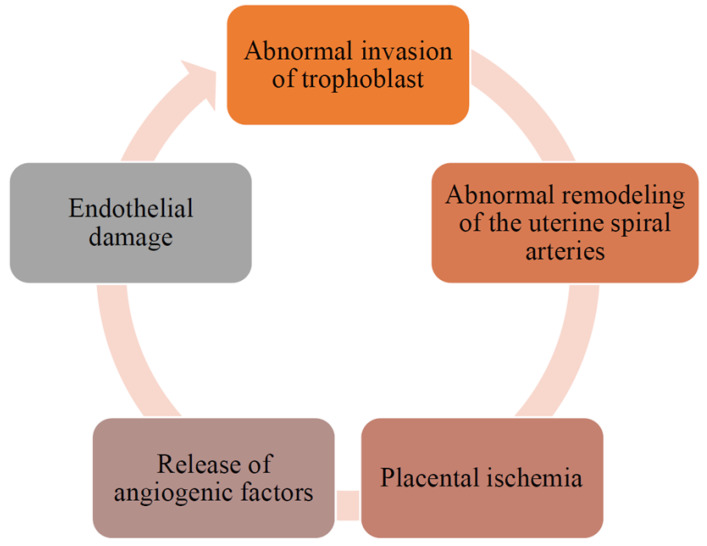
Pathogenesis of preeclampsia.

Although the pathogenesis of preeclampsia is not yet fully understood, the main pathological mechanisms have been clarified [1,2,5,6]. The main hypothesis explaining preeclampsia development is a defective trophoblastic invasion with associated utero-placental hypoperfusion [1,2,5,6,86]. Based on the available evidence, a two-stage model of preeclampsia pathogenesis was developed: (1) abnormal remodeling of the uterine spiral arteries leading to placental ischemia; (2) the release of antiangiogenic factors by the ischemic placenta into the maternal circulation with the subsequent development of endothelial damage (Figure 2) [1,2,4,6]. Multiple factors are involved in the pathogenesis of preeclampsia: vascular endothelial growth factor (VEGF), transforming growth factor beta (TGF-β), placental growth factor (PlGF), soluble FMS-like tyrosine kinase 1(sFlt-1) and soluble endoglin (sEng) nitric oxide (NO), angiotensin receptor 1 antibodies (AT1-AAs), etc. [1,2,5,6]. These factors equally contribute to the development of gestational hypertension, preeclampsia, and IUGR [2]. However, this two-step theory does not explain the specific manifestations of preeclampsia (symptoms and signs), which are different from gestational hypertension and/or IUGR [2,6]. Thus, preeclampsia is always caused by compromised placental perfusion; however, other risk factors contributing to preeclampsia remain unclear [2,6,8]. Authors should discuss the results and how they can be interpreted from the perspective of previous studies and of the working hypotheses. The findings and their implications should be discussed in the broadest context possible. Future research directions may also be highlighted.

#### 3.3.3. The Role of MTHFR Polymorphism and Hyperhomocysteinemia in Preeclampsia

**Figure 3:**
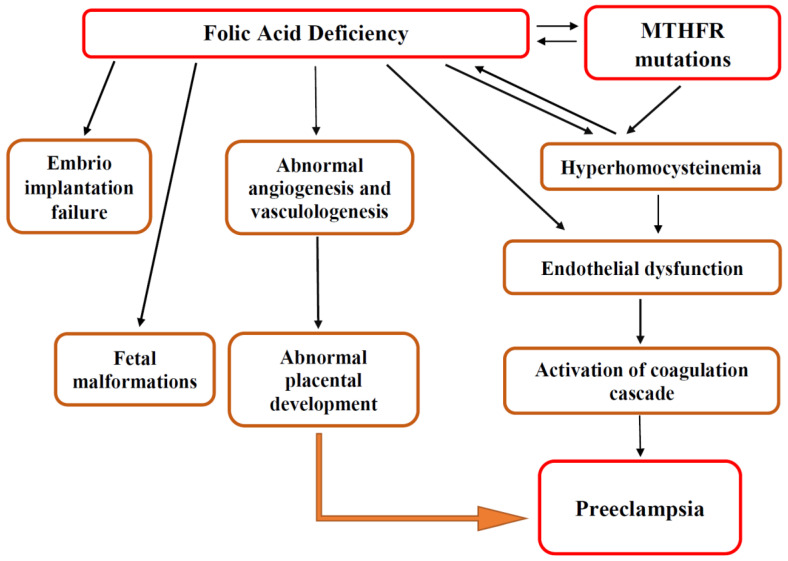
Role of folic acid and *MTHFR* polymorphisms in preeclampsia. Figure legend: MTHFR—methylenetetrahydrofolate reductase.

Pregnancy is associated with major physiological and immunological changes in a woman’s organs and organ systems, which are controlled by “methylation patterns of certain candidate genes” [87]. The *MTHFR* gene is one of the genes taking a pivotal role during pregnancy by virtue of thrombotic events or methylation [87]. The *MTHFR C677T* polymorphism results in low enzymatic activity and an accumulation of homocysteine [87]. Hyperhomocysteinemia leads to thrombotic events in a vascular bed of various localizations, including the placenta, causing severe conditions (Figure 3) [14,27,29,33,52,53,54].

Increased blood levels of homocysteine during pregnancy are associated with multiple pregnancy complications, including preeclampsia, spontaneous abortions, IUGR, and placental abruption [54,88,89,90]. Moreover, elevated plasma homocysteine has been proposed to be an independent risk factor for preeclampsia [88,89,90]. Hyperhomocysteinemia results in the induction of inflammatory determinants including the expression of adhesion molecules, leukocyte adhesion, and endothelial cells damage [42,54]. In turn, endothelial injury promotes oxidative stress, reduces NO bioavailability, and increases the production of vascular procoagulants [42,54,86]. Further, endothelial damage leads to decreased protein C activation, which further contributes to local pro-coagulation.

Several case–control studies and systematic reviews have reported that hyperhomocysteinemia is associated with preeclampsia [13,54,91,92,93,94,95]. These studies found the average homocysteine levels to be significantly higher in women with preeclampsia compared to healthy controls (*p* < 0.001). Furthermore, Acilmis et al., (2011) reported that the maternal and fetal homocysteine blood levels were significantly higher in patients with severe preeclampsia compared to preeclampsia and healthy controls [94]. These data support the hypothesis that hyperhomocysteinemia might be associated with the severity of preeclampsia [94].

Moreover, recent studies reported that the *MTHFR C677T* gene polymorphism seems to play a significant role in preeclampsia [87], thus contributing to the hyperhomocysteinemia state. These findings suggest that folic acid supplementation in women could decrease the level of serum homocysteine and, therefore, reduce the risk/severity of preeclampsia (Figure 3) [8,13].

### 3.4. Folic Acid Supplementation for Preeclampsia Prevention

As placental development is a cornerstone in preeclampsia pathogenesis, the placental growth processes take special attention. Placental development and circulation require angiogenesis, which is critical for the development of a physiological placental blood flow and normal development of the fetus (Figure 3) [2,5,6,38]. Folic acid plays an essential role in this process via induced angiogenesis, through a NO-dependent mechanism [38]. Thus, folic acid supplementation for preeclampsia prevention remains a hot topic for researchers’ discussions and investigations.

Studies report that adequate folic acid supplementation from the onset of pregnancy supports physiological trophoblastic proliferation and angiogenesis and helps avoid increased levels of homocysteine that can be seen in the third trimester [4,40,88]. Several investigations’ results suggest that folate supplementation is beneficial for the reduction of preeclampsia and gestational hypertension incidences (Table 1) [8,12,22,38,96,97,98,99,100,101,102,103].

First-trimester folate-only supplementation (not multivitamins) has been associated with a reduction in the risk of preeclampsia (AOR 0.42, 95% CI: 0.13, 0.98) [99]. A study by Wang et al., (2015) on the Chinese population who followed the dietary intake of folate during pregnancy was associated with a reduced risk of severe preeclampsia (OR: 0.52, 95% CI: 0.31, 0.87) with a significant dose-related response, but not in mild preeclampsia [98]. A Canadian prospective study reported an association of ≥1.0 mg folic acid supplementation with a lower rate of preeclampsia, “and the results were statistically significant in women with an increased risk of developing preeclampsia” [97]. These studies support the hypothesis that folate supplementation and dietary folic acid intake during pregnancy are associated with a reduced risk of preeclampsia.

High doses (>1.0 mg/day) of folic acid intake and prolonging the period of folate supplementation beyond the first trimester of pregnancy was proposed to aid in preeclampsia prevention through the support of physiologic angiogenesis [4,38,95]. Some authors investigated the possible role of folic acid in the regulation of trophoblastic invasion and the development of the placenta [38,40]. The beneficial effect of extended folate administration through a pregnancy course for a reduced incidence of preeclampsia was suggested [38,95,96]. Moreover, longer-term folic acid supplementation in pregnancy was proposed to reduce the secondary systemic symptoms of preeclampsia due to its favorable effects on endothelial function [38].

However, others demonstrated no association between folic acid supplementation and preeclampsia [4,8,20,23,24,104,105]. A study on the Danish population fail to show that folate was related to preeclampsia risk [23]. The same conclusion was made by researchers from Columbia who found that prenatal consumption of folic acid did not decrease the risk of preeclampsia in the Columbian population [105]. A recent systematic review, which aimed “to investigate the effect of maternal folic acid supplementation during pregnancy on the risk of preeclampsia and gestational hypertension” reported little evidence for the association between maternal folic acid administration and decreased risk of preeclampsia [4,20]. In addition, the FACT trial, a double-blind, phase III, randomized controlled, multicenter study did not show benefits from prolonged supplementation with 4.0 mg/day of folic acid for preeclampsia prevention [21]. The most recent study on the effect of high-dose (4 mg/day throughout pregnancy) folic acid supplementation for preeclampsia prevention in twin pregnancies did not show any benefits [106].

However, since increased levels of homocysteine lead to endothelial dysfunction (thus associated with preeclampsia pathogenesis), and taking into consideration the fact that 5-MTHF is the active form of folate directly involved in the metabolism of homocysteine into methionine, folate administration during pregnancy could prevent hyperhomocysteinemia and play an important role in preeclampsia prevention [4,38,94]. Moreover, 5-MTHF was reported to have the ability to stimulate the endothelial production of NO, which has anticoagulant activity through vasodilatatory and antiplatelet functions [4,107,108,109]. Therefore, in spite of the controversies reported about the role of folic acid supplementation for preeclampsia prevention, there is a pathophysiological background behind the continuous investigations in this field.

The genetic background of racial and ethnic diversity is under continuous investigation in modern genetics, and studies of the human populations’ genetic structure have found variations between racial and ethnic groups [72,73,74]. The fact that there is a disparity in preeclampsia prevalence among different racial and ethnic groups [72,73] could suggest specific pathogenetic pathways of the condition. Thus, investigations to evaluate folic acid supplementation for the prevention of preeclampsia should take into consideration the genetic and ethnic background of study participants, doses, and periods of folic acid administration. Therefore, further studies are required to outline the protocol for effective folic acid supplementation in women to prevent preeclampsia.

## 4. Conclusions

The essential role of folate supplementation in pregnancy for neural tube defect prevention is doubtless. However, the association between the dosage of folic acid supplementation and the potency of its effect in other pregnancy-related conditions should be clarified. Further investigations to evaluate the opportunity of folic acid supplementation for preeclampsia risk reduction should take into consideration the dose-dependent effects, duration of administration, and ethnic background of patients. Therefore, more case–control studies need to be conducted to clarify the role of folic acid supplementation in preeclampsia prevention.

## Figures and Tables

**Table 1:** Folic acid supplementation for preeclampsia prevention.

Authors	Population	Recommendations		Reference
		Dose	Time and Duration	
Manizheh et al., 2009	Iranian	Folic acid, 500 μg/day–5 mg/day	From early pregnancy until delivery	[12]
Kim et al., 2014	South Korean	Folic acid, 400 μg/day–1.0 mg/day daily for at least	Two-three months before conception and throughout pregnancy	[101]
Martinussen et al., 2015	USA	Folic acid, <200 μg/day, 200 < 600 μg/day, ≥600 μg/day	One month before pregnancy, first-trimester pregnancy	[102]
Wang et al., 2015	Chinese	Dietary folic acid intake (from 151.6 μg/day to ≥274 μg/day)	Throughout the whole pregnancy	[98]
Vanderlelie et al., 2016	Australian	Folic acid, 800 μg/day	The first trimester of pregnancy	[99]
Wen et al., 2016	Canadian	Folic acid, ≥1.0 mg/day	First and early second trimester of pregnancy	[97]
Han et al., 2020	Chinese	Folic acid, <400, 400, and >400 μg/day	Before and during pregnancy	[100]
Zheng et al., 2020	Chinese	Folic acid, 4 mg/day	Three months before pregnancy for the entire pregnancy	[103]

## Data Availability

Data related to this article could be retrieved from authors (gulzhanat.aimagambetova@nu.edu.kz) per reasonable request.
